# The Structure–Function Linkage Database

**DOI:** 10.1093/nar/gkt1130

**Published:** 2013-11-23

**Authors:** Eyal Akiva, Shoshana Brown, Daniel E. Almonacid, Alan E. Barber, Ashley F. Custer, Michael A. Hicks, Conrad C. Huang, Florian Lauck, Susan T. Mashiyama, Elaine C. Meng, David Mischel, John H. Morris, Sunil Ojha, Alexandra M. Schnoes, Doug Stryke, Jeffrey M. Yunes, Thomas E. Ferrin, Gemma L. Holliday, Patricia C. Babbitt

**Affiliations:** ^1^Department of Bioengineering and Therapeutic Sciences, University of California, San Francisco, San Francisco, CA 94158, USA, ^2^Universidad Andres Bello, Center for Bioinformatics and Integrative Biology, Facultad de Ciencias Biologicas, Santiago 8370146, Chile, ^3^Nodality, Inc., South San Francisco, CA 94080, USA, ^4^Department of Electrical and Computer Engineering, College of Engineering, Boston University, Boston, MA 02215, USA, ^5^Department of Chemical Engineering, Massachusetts Institute of Technology, Cambridge, MA 02139, USA, ^6^Department of Pharmaceutical Chemistry, School of Pharmacy, University of California, San Francisco, San Francisco, CA 94158, USA, ^7^Center for Bioinformatics (ZBH), University of Hamburg, Hamburg 20146, Germany, ^8^Department of Chemistry and Biochemistry, Montana State University, Bozeman, MT 59717, USA, ^9^School of Medicine, University of California, San Francisco, San Francisco, CA 94143, USA, ^10^UC Berkeley - UCSF Graduate Program in Bioengineering, University of California, San Francisco, CA 94158 and Berkeley, CA 94720, USA and ^11^California Institute for Quantitative Biosciences, University of California, San Francisco, San Francisco, CA 94158, USA

## Abstract

The Structure–Function Linkage Database (SFLD, http://sfld.rbvi.ucsf.edu/) is a manually curated classification resource describing structure–function relationships for functionally diverse enzyme superfamilies. Members of such superfamilies are diverse in their overall reactions yet share a common ancestor and some conserved active site features associated with conserved functional attributes such as a partial reaction. Thus, despite their different functions, members of these superfamilies ‘look alike’, making them easy to misannotate. To address this complexity and enable rational transfer of functional features to unknowns only for those members for which we have sufficient functional information, we subdivide superfamily members into subgroups using sequence information, and lastly into families, sets of enzymes known to catalyze the same reaction using the same mechanistic strategy. Browsing and searching options in the SFLD provide access to all of these levels. The SFLD offers manually curated as well as automatically classified superfamily sets, both accompanied by search and download options for all hierarchical levels. Additional information includes multiple sequence alignments, tab-separated files of functional and other attributes, and sequence similarity networks. The latter provide a new and intuitively powerful way to visualize functional trends mapped to the context of sequence similarity.

## INTRODUCTION

As current technologies generate an immense number of protein sequences, the size of primary databases is increasing at an exponential rate (1). As a result, the characterization of protein function lags increasingly behind data availability. This gap serves as a main motivation for development of computational methods for protein classification and function assignment, along with making these data accessible and useful. To a large extent, functional inference methodologies rely primarily on protein sequence similarity as a proxy for function similarity, although it is clear that addition of other types of information improves the results (2). Indeed, protein domain databases such as FunTree (3), PIRSF (4), SUPERFAMILY (5), ClusTr (6), TIGRFAMs (7), SUPFAM (8), InterPro (9), Pfam (10), Gene3D (11) and the Conserved Domain Database (12) utilize this principle, sometimes combined with structural information, to bin proteins into classes associated with particular functions.

Because enzymes catalyze chemical transformations, the mapping between sequence features and the molecular roles they perform can be explicit, so that the chemistry they catalyze provides an alternative way to describe their relationships. Thus, other researchers classify enzymes using approaches that are complementary to sequence-based groupings. Examples include the Enzyme Nomenclature Classification (EC) system (13,14), MACiE (15), Catalytic Site Atlas (16), EzCatDB (17), KEGG (18) and BRENDA (19), in which the main grouping criteria are derived from their chemical reactions and/or associated ligands.

Sequence- and chemical-centric approaches for enzyme classification are limited, however, because sequence–function correspondence is complex and variable. Generally, there is no single sequence similarity threshold for accurately grouping sequences into isofunctional groups. This makes it hard to delineate sequence-similarity boundaries to identify proteins that carry out the same function or chemical-similarity (of the substrate or reaction) boundaries that coincide with sequence-based groupings. This results in, on the one hand, high rates of misannotation (20) and, on the other hand, the definition of many protein groups that cannot be assigned a *specific* function. One approach that attempts to integrate these conflicting concepts is based on the idea of ‘functionally diverse’ (or ‘mechanistically diverse’) protein superfamilies (21,22). Members of these types of superfamilies can be highly divergent and catalyze quite different overall reactions. Nevertheless, all members of such an enzyme superfamily must share a tenable evolutionary ancestor, some conserved aspect of chemistry, and active site features. For example, members of the superfamily might catalyze the same partial reaction or stabilize the same type of intermediate using a characteristic set of conserved residues. In many cases, this set of residues is only a subset of those responsible for the overall reaction; other residues are frequently used as specificity determinants. Notably, there are cases in which superfamilies are grouped into a broader *suprafamily* (22), a set of evolutionarily related enzymes whose members share similar active site architecture but utilize this conserved architecture in substantially different ways. Previous estimates for the number of functionally diverse superfamilies suggest that they represent over a third of known enzyme superfamilies (23). This suggests the wide applicability of grouping enzymes based on common ancestry and shared active site features, even when they catalyze different overall chemical reactions.

**Figure 1.**
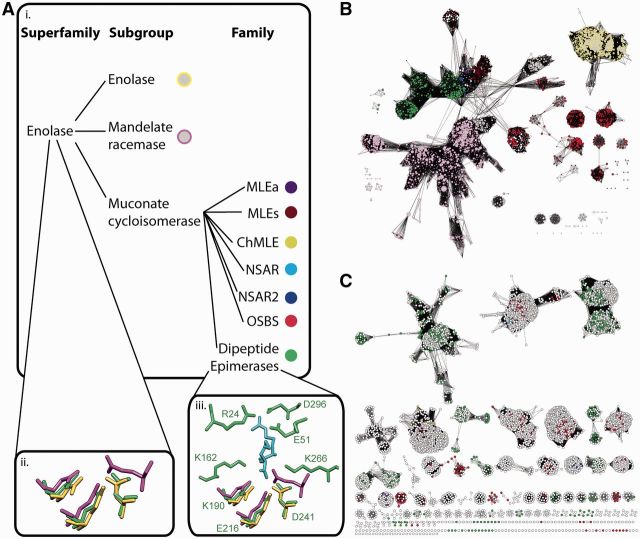
Hierarchical classification in the SFLD, SSNs and their potential contribution for enzyme classification and function prediction. (**A**) SFLD classification is exemplified by the enolase superfamily. (i) This superfamily is divided into seven subgroups; three of them—the enloase, mandelate racemase and muconate cycloisomerase subgroups—are shown in this panel. As shown here for the muconate cycloisomerase subgroup only, these subgroups are divided into families. (Note: The same name, e.g. enolase, can represent a superfamily, a subgroup, and a family.) Colored circles serve as a legend for panels B and C. (ii) Superposition of three residues reflecting conservation of important active site machinery across all members of the superfamily. Each color represents a different structure, one from each of the three subgroups: a dipeptide epimerase in green (PDB: 3RIT), a mandelate racemase in magenta (PDB: 1MDR) and an enolase in yellow (PDB: 7ENL). All enolase superfamily members share three metal binding active site residues that participate in a common partial reaction, abstraction of proton, that initiates each of their different overall reactions. (iii) Dipeptide epimerases, members of a family within the muconate cycloisomerase subgroup, share functionally important residues—three conserved in all members of the superfamily and associated with the proton abstraction, and two additional residues (K162, K266) that also contribute to proton abstraction. Another set of residues (upper part of panel iii, R24, E51 and D296) are thought to participate in the specificity of some dipeptide epimerases (35). Thus, these latter three residues differentiate these dipeptide epimerases from other families in the superfamily. The dipeptide ligand crystallized with 3RIT is shown in cyan. (**B**) A representative SSN of the enolase superfamily. Each node represents all sequences that share >70% sequence identity. Node size corresponds to the number of sequences that are represented by the node; the smallest nodes represent one sequence, and the largest nodes represent >100 sequences. Edges between representative nodes indicate a mean BLAST *E*-value, between all pairs of sequences in these nodes, <1e^−43^. Coloring is as shown in subgroup (node border color) and family (node fill color) sub-panels in A (**C**) A full SSN of the muconate cycloisomerase subgroup. Each node represents a single protein, and each edge indicates a BLAST *E*-value <1e^−80^. Using this network representation layout, within-cluster similarities are greater than similarities between clusters. Nodes are colored only if they are associated with reliable evidence, i.e. better evidence than ‘inferred from electronic annotation’. Different families are color-coded (panel A). The correspondence between function and sequence is evident in the network. Different families tend to appear in specific sequence clusters, allowing reliable (and visual) delineation of the sequence space that corresponds to a specific function.

Here, we describe the Structure–function Linkage Database (SFLD) (24,25). This resource focuses on the identification of specific sequence and structure attributes reflected in protein similarity that typify specific reactions or substrate specificities. It links enzyme sequence, structure and molecular function into a hierarchical classification scheme, grouped by enzyme superfamilies. Each superfamily in the SFLD is subdivided into successive levels of increasing specificity in sequence similarity and associated functional properties. One or more levels of subgroups within a superfamily represent distinct subsets defined principally by sequence similarity. Below the subgroup level, enzyme families represent the finest classification level, in which each member is annotated to catalyze the same reaction using the same mechanistic strategy (Figure 1, panel A).

The SFLD is based upon a relational database implemented in MySQL (http://www.mysql.com/) that follows a carefully designed schema. The web interface is implemented in the Django Application Programming Interface (https://www.djangoproject.com/) and is professionally maintained and extended. This interface enables both presentation of the information for outside users and data manipulation and updating by curators, while ensuring data integrity.

The examination of the similarity relationships between sequences for purposes like classification and function prediction can be greatly facilitated by a unique and powerful tool: protein sequence similarity networks (SSNs) (26–28), in which nodes represent proteins and edges represent sequence similarities between them, as measured by even simple metrics such as BLAST *E*-values (29). Networks provide an intuitively accessible visualization infrastructure for exploration of sequence–function relationships and can be used as an effective complement to traditional methods such as multiple sequence alignments (MSAs) and phylogenetic trees (26,28). SSNs are fast to generate and although still subject to size limitations, they can still comprise many thousands of sequences. Thus, they can produce a global summary of sequence similarities across even large superfamilies. Furthermore, they can be manipulated to aid protein classification and function prediction. For instance, ‘coloring’ known functional attributes onto the network nodes visually highlights which proteins of unknown function may share similar functional attributes. Modification of the similarity threshold required for displaying network edges enables alternative hypotheses for partitioning superfamily members (Figure 1, panel B). The usage of SSNs in the SFLD is supported by Pythoscape (30), a recently described infrastructure for creating, manipulating and integrating these networks. SSNs can be downloaded from the SFLD for all levels of the classification hierarchy and analyzed locally using Cytoscape (31), a freely available cross-platform program for complex network analysis and visualization.

## DATABASE CONTENT

### Classification

The SFLD is designed to make the hierarchical classification of enzyme superfamilies and their associated data accessible to users for many applications in function prediction, tracking evolutionary trends and providing guidance for enzyme engineering. It includes sophisticated tools for browsing the sequence hierarchy and list of chemical reactions. Searching can be done by sequence or several different external identifiers [e.g. UniProtKB (1) accession numbers]. The basic classification unit in the SFLD hierarchy is an ‘enzyme functional domain’ (EFD), namely a consecutive set of residues that may or may not span the whole protein and is responsible for a biochemical function. This means that if a single protein includes multiple distinct domains that carry out different chemical reactions, each EFD will be assigned to the appropriate level(s) of the hierarchy, with each EFD perhaps belonging to a different superfamily. Frequently, an EFD will be associated with a family; but when an EFD family assignment cannot be determined, it is assigned only at a broader level, to a subgroup and/or a superfamily.

EFDs are calculated for proteins as they are added to the database based on their matches to Hidden Markov Models (HMMs) associated with SFLD superfamilies, subgroups and families. These sequence signatures are calculated from an MSA of canonical members of the relevant subgroup or family using the HMMER (32) package. Eighty percent of the families and 60% of the superfamilies in the SFLD are accompanied by a manually curated MSA and the corresponding HMM, depending on the depth at which a superfamily is curated. EFDs in the SFLD are also annotated with external database identifiers, statistics such as length, taxonomy and additional functional information.

### Main database categories: core and extended SFLD

The core SFLD comprises 12 manually curated functionally diverse superfamilies in which known functional attributes have been transferred to over 345 000 proteins (Table 1). We consider the most highly curated superfamilies in the core SFLD as a ‘gold standard dataset’, especially intended for purposes like training and evaluating automated methods for function prediction. This set (33) has been used by us and others for cataloguing misannotation in major public databases (20).

**Table 1.** General statistics for data stored in the SFLD

Superfamily	Subgroups	Families	Sequences	Structures	Reactions
Core SFLD					
Amidohydrolase	11	89	36 690	385	42
Aromatic Prenyltransferase	2	0	339	17	0
Crotonase	2	27	41 143	162	28
Enolase	7	20	23 460	341	22
Glutathione Transferase (cytosolic)	42	0	12 904	400	0
Haloacid Dehalogenase	25	22	79 778	515	21
Isoprenoid Synthase Type I	14	69	16 385	298	65
Isoprenoid Synthase Type II	4	8	7635	179	8
Nucleophilic Attack 6-Bladed Beta-Propeller (N6P)	3	3	31 085	69	2
Peroxiredoxin	6	0	12 239	154	0
Radical SAM	52	84	48 100	40	60
RuBisCO	2	2	36 390	69	2
Total	170	324	348 859	2629	250
Extended SFLD^a^					
Six-hairpin glycosidases	0	0	28 690	214	0
Ribulose-phosphate binding Barrel	0	0	25 997	241	0
Metalloproteases, Zincins	0	0	21 437	420	0
Cytidine deaminase-like	0	0	18 803	91	0
l-Aspartase-Like	0	0	17 659	99	0
Carbon–Nitrogen Hydrolase	0	0	14 974	44	0
Carbohydrate Phosphatase	0	0	12 278	149	0
Phosphatidylinositol Phosphodiesterase	3	5	11 014	95	5
Arginase/Deacetylase	0	0	10 570	168	0
Fumarylacetoacetase, C-terminal-related	0	0	10 104	31	0
Ferric Reductase Domain	2	2	282	1	0
…	…	…	…	…	…
Total^b^	9	11	437 633	4288	9

^a^A representative set from the extended SFLD (34 superfamilies at the time of publication).

^b^The total counts refer to the whole extended SFLD set.

To address the limits of manual curation in achieving broader coverage of the superfamily universe and to keep pace with the increasing size of the sequence data from genomic and community sequence projects, we have instituted the ‘extended’ SFLD, aimed at automatic structure–function mapping of sets of related proteins via SSNs (Table 1). These sets are not restricted to functionally diverse superfamilies, although many fall into this category, consistent with the focus of the SFLD. The extended SFLD provides a shallower level of curation than the core; very few entries include any detailed functional annotation or subgroup/family assignments, except as can be obtained from automatically retrieved links to other public databases. Nevertheless, continuous efforts to manually curate this section of the SFLD will eventually move some functionally diverse superfamilies from the extended SFLD to the core set. SSNs in the extended SFLD provide summaries of sequence relationships associated with many types of functional properties. Because these networks can be produced using automatic protocols they increase the number of superfamilies for which some structure–function mapping can be provided. SSNs have been valuable in providing clues for functional inference in superfamilies [e.g. (34–36)]. Hence, users who are interested in enzymes not covered by the core SFLD (see Common Use Cases below) may utilize these networks for hypothesis generation and function prediction. Examples of some biologically interesting superfamilies included in the extended SFLD are listed in Table 1. These superfamilies include enzymes associated with human diseases and disease-related polymorphisms, or encoded in human pathogenic bacteria. New superfamilies are added more often to the extended SFLD than to the core SFLD. As a result, the list of extended SFLD superfamilies for which networks are available is expected to increase frequently relative to addition of new superfamilies to the core SFLD. The SFLD infrastructure and data are extended and updated regularly. At some times, full or representative networks and other data may not be available as a result. Users should address questions to the Help Desk.

### Database curation

The first step in manual superfamily curation for the core SFLD is the collection of a set of protein sequences and creation of a hierarchical classification utilizing the SFLD’s curator interface. Collecting and analyzing the data requires a substantial level of chemical knowledge, as the curator must identify the partial reaction or other chemical capabilities that serve as a common feature conserved among all superfamily members. Starting with published collections, where sources may include reviews, personal communications, sequence pattern databases [e.g. InterPro, Pfam, SCOP (37) or CATH (38)] and Enzyme Commission (EC) information, the curator collects a set of proteins that show preliminary agreement with our definition of a superfamily. This draft set is used by the SFLD protocol for automatic retrieval of functional attributes from various databases. In addition to these tabulated data, the SFLD can produce an SSN (Figure 1, panels B and C) in which nodes have been assigned various automatically retrieved annotations. This allows the curator to research the correspondence between sequence similarity and functional similarity in the draft superfamily. Manual classification into subgroups and families is subsequently done using SSN analysis and the curator’s interface. This includes incorporation of structural information, MSAs, active site residues, chemical reactions and partial reactions, and descriptions of enzymatic mechanisms based on the literature. Finally, for all core SFLD superfamilies, the curation is often informed through collaborative work with enzymologists who are experts in the structure–function relationships of these superfamilies.

### Evidence codes

Each of the annotation features associated with proteins that appear in the SFLD are accompanied by evidence codes. These codes describe the rationale and reliability behind the classification and annotation assignments. They are required for evaluating the reliability and validity of curation, allowing more accurate function prediction for query proteins (see Common Use Cases). Data in the SFLD represent many types and resources, ranging from annotations from external databases, to assignments of conserved residues, families, subgroups and superfamilies, to partial reaction steps. This range of evidence necessitated creating a vocabulary of evidence codes that are widely used and understood [following Gene Ontology (39) definitions], such as ‘Inferred from Electronic Annotation’ and ‘Inferred from Direct Assay’, or SFLD specific (e.g. ‘Canonical Family Member’ or ‘Inferred from Catalytic Residues’). Descriptions of all evidence codes and data types can be found in the online documentation of the database.

## BROWSING AND SEARCHING

The SFLD offers two distinct entry points for browsing the classification hierarchy: ‘Browse by Superfamily’ and ‘Browse by Reaction’. Data searching can be accessed via the ‘Search by Enzyme’ mechanism. These data gateways offer the means to answer key user inquiries.

### Browse by superfamily

This entry point to the hierarchy allows users to study a superfamily of interest at several levels of granularity. Upon selecting a ‘core’ or ‘extended’ SFLD superfamily, the superfamily’s summary page is displayed, providing a general description and relevant references, as well as statistics such as the numbers of sequences, structures and reactions currently associated with that superfamily in the database. Users can easily navigate to the summary pages of subgroups and/or families within the superfamily, and at even finer levels, individual EFDs, structures and chemical reactions.

The structure of subgroup and family pages is similar to that of the superfamily summary page. Each level includes download options for the SSNs (discussed in more detail below) and tab-separated annotation files, along with table views that can be customized (columns can be interactively added or removed). In addition, users may filter the sequences associated with a family/subgroup/superfamily to specific organism/s of interest. To enable examination of conservation within superfamily members, users can choose to view the manually curated MSA, if available, of representative members of the superfamily, subgroup or family. Curators choose representative sequences for the MSA to avoid bias in sequence representation and to ensure broad coverage of sequence diversity. Importantly, the positions of the conserved catalytic residues, where known, are highlighted in the alignment, and an accessory table lists the roles of these residues (‘metal binding’ etc.). An additional useful tool allows users to align a query protein or set of proteins to the MSA, when available.

A family page lists the overall chemical transformations that are catalyzed by family members (including reactants) and provides links to external databases such as EC, IntEnz, MetaCyc (40) and BRENDA. This page also includes an image of the active site and family-conserved functional residues in a representative protein structure (if available). Clicking the active site image downloads the corresponding session file for interactive viewing and analysis in UCSF Chimera (41), which must be installed locally on the user’s computer. Chimera is free for noncommercial use; it is available for all major platforms and includes full documentation and tutorials. The MSAs mentioned above can also be viewed in Chimera, along with or independent of associated structures, which enables calculating different measures of sequence conservation, mapping conservation in the alignment onto structures and many other types of analysis.

Importantly, an additional entry point to superfamily browsing is structure-centered. Each SFLD superfamily has a table that lists the relevant protein structures, along with all EFDs that share 95% identity with a structurally solved protein. Related data such as resolution, HET groups and whether the protein is mutated are also supplied, along with additional links [to the Catalytic Site Atlas, PDBSum (42) and the RCSB PDB (43)].

### Full and representative SSNs

For those not limited by size, full SSNs can be downloaded for each superfamily in the SFLD. These networks can be opened and manipulated using locally installed Cytoscape software. In full SSNs, each node represents a unique protein sequence, and each edge represents the pairwise similarity between the connected nodes. The network view summarizes structure–function mappings for large sequence sets (as in some superfamilies or subgroups within them), while allowing the user to examine individual sequences, their functional attributes and their similarity relationships at finer levels of the hierarchy. All of the annotations available for each EFD are included as node attributes in the networks (e.g. useful attributes like family/subgroup membership, species, and, for cloning or microbiology studies, availability of DNA). The display properties of the nodes in Cytoscape can be changed to indicate attribute values. For example, nodes could be colored by taxonomy and nodes that represent proteins associated with a PDB structure could be shown with a different shape.

SSNs are currently provided in XGMML format and packaged with a README file describing the download contents, and an attribute file customized for the superfamily. Another feature under development (and already available for some SSN downloads) is the inclusion of statistical details specific to the network, such as protein length distributions and plots depicting the dependency between *E*-value thresholds and SSN edge numbers. For full networks, the download form allows the user to specify the threshold for edge inclusion, i.e. the minimal level of similarity required for displaying edges between sequences. Setting the threshold at more stringent similarity values can enable viewing of larger networks as the number of edges governs the size limits at which networks can be opened on a particular computer.

Whereas full networks allow detailed analysis, there is an upper limit to the number of edges that can be viewed in the network, due to computer memory limitations (>250 000 edges will significantly slow down a standard laptop running Cytoscape). Thus, we are now transitioning to produce ‘representative’ SSNs (30) for these sequence sets. In a representative network, each node represents a set of proteins that share a curator-defined sequence identity level and each edge represents the mean similarity between the connected nodes. These networks can provide a global snapshot of the sequence space of an entire superfamily, even tens of thousands of sequences, owing to this method for abstracting the data. This facilitates the elucidation of global trends and sequence space topology for large sets of enzymes and can be followed by a more detailed analysis using full SSNs for smaller sets of proteins such as subgroups. Compared to full networks, the assignment of node attributes to representative SSNs is more complex since an individual node can represent multiple sequences. Thus, only a subset of the node attributes in full SSNs are available in representative SSNs (see online documentation).

### Browse by reaction

Another entry point to the SFLD allows browsing by reaction. This web page includes a total of 251 chemical reactions, accompanied and sorted by EC numbers, if available, for each reaction. Additional information includes the associated superfamily/family for each reaction and the number of EFDs annotated to catalyze the reaction.

### Search by enzyme

The ability to search by enzyme is described below using several different questions that database users may ask:

‘Is my query protein sequence present in the SFLD, or, if not present, similar to an SFLD entry?’ The ‘search by sequence’ option provides a sequence similarity search against the SFLD database. The default search option, ‘by BLAST’ will generate two tables describing the search hits. The first table lists the superfamilies, subgroups and families that the hits belong to, as well as statistics like the number of hits and the range of *E*-values and scores for these hits. Notably, if the hit is already in the SFLD, then an SSN will be made available for the subgroup and/or family to which the sequence belongs. The query node in the SSN download will be highlighted, allowing the user to see it in the wider context of the family or subgroup. The second is a downloadable table of SFLD entries, accompanied by BLAST similarity measures (*E*-value, score, alignment length), EFD attributes and an option to display the pairwise alignment between the query and the SFLD entry. Alternatively, choosing the ‘by HMM’ search option will run all the HMMs present in the SFLD against the query sequence, and output a downloadable table of statistically significant matches of families, subgroups or superfamilies. For each hit, the user can also align the query sequence with the relevant MSA. The analysis of the MSA is facilitated by the highlighted active site positions in the aligned sequences and the query protein.

‘Can I find an existing SFLD entry using commonly used protein identifiers and names?’ The SFLD can be searched using external protein identifiers (single or combinations of GI numbers, UniProtKB accessions, etc.) or structure identifiers (PDB ID), or internal identifiers (EFD ID) or EFD name. The name allows users to find a specific protein, but also to find any proteins sharing protein name parts. The result table that is generated by this search can then be sorted and filtered by the user.

‘Can I find all SFLD entries associated with a specific organism?’ Typing a scientific species name or names will trigger a mechanism that retrieves all SFLD entries that are mapped to that query species.

## DATABASE INFRASTRUCTURE

### Automatic maintenance

A fundamental requirement for high-quality databases is a reliable maintenance mechanism that will keep identifiers and links up to date. Python scripts keep the SFLD updated by periodically checking references to external databases (e.g. verifying identifiers and links). In addition, the RCSB PDB database is queried for new and relevant protein structures. Notably, SSNs will be periodically updated to reflect the current protein repository of the database. SFLD EFD entries post the last date on which they were manually or automatically checked.

### Documentation and tutorials

The SFLD database includes substantial documentation and tutorial sections, providing both term definitions and detailed protocols for database usage. The documentation includes a glossary of terms, an evidence code table and a description of the SSNs in general, along with a detailed description of the SSNs provided by the SFLD (including a list of the 30 SSN node attributes and three edge attributes). The tutorials include video introductions with an overview of the database and a step-by-step example describing characterization of a protein of unknown function. A video that introduces SSNs and their use for function prediction is also available.

## COMMON USE CASES

The combination of the information available from the SFLD via the web interface and the SSN downloads offer a powerful tool for function prediction, discovering misannotations and the selection of new proteins for structural or functional characterization (‘target’ selection). A few examples are provided below.

### Function prediction and addressing misannotation

The sequence of the protein GI:390523686 from *Desulfitobacterium dehalogenans* originates in a whole-genome sequencing study. According to the NCBI database, the protein is annotated as ‘l-lysine 2,3-aminomutase’. Of the top 100 BLAST hits (against NCBI NR), 91 share the same name. Notably, 97 of the 100 hits share 42–46% sequence identity with the query protein.

**Figure 2.**
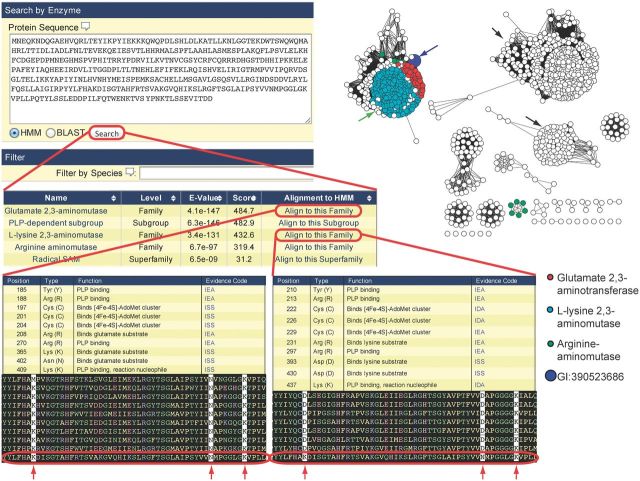
Searching by sequence in the SFLD. The screenshot at the top shows the query sequence, GI:390523686. Choosing ‘search’ (with the HMM option) compares all SFLD HMMs against the query sequence. The table of results (middle panel) lists all the classification levels (family/subgroup/superfamily) for which a relevant hit was found. The two top families are glutamate 2,3-aminomutase and l-lysine 2,3 aminomutase. Clicking the ‘Align to this family’ button leads to a list of the active site residues that appear in the family, and to an MSA of the family with the query included as the bottom sequence (red ellipse). As explained in the text, although the protein is annotated in GenBank as l-lysine 2,3 aminomutase, evaluation of the MSA suggests that the characteristic active site residues for that function are not conserved in the query (indicated by the red arrows below the bottom panel). Instead, this analysis supports the annotation of this sequence as a glutamate 2,3-aminomutase. The user can also download an SSN of the relevant subgroup, here thresholded at 1e^−85^, which includes the two above-mentioned families. In the figure, l-lysine 2,3-aminomutases are shown in cyan, glutamate 2,3-aminomutases in red and arginine aminomutases in green. The query protein is represented by a blue circle and arrow. This network perspective also supports the annotation of the query protein as a glutamate 2,3-aminomutase. Two examples of clusters that do not include any annotated proteins are indicated by black arrows, hinting at potentially new functional families in this subgroup. A few of the white-colored sequences of unknown function that can potentially be annotated as l-lysine 2,3-aminomutases are indicated with a green arrow.

When using the ‘search by enzyme’ (‘HMM’ option), the user is presented with the most probable family assignments for this query protein (Figure 2). The highest-scoring family is glutamate 2,3-aminomutase, followed by l-lysine 2,3-aminomutase (these families belong to the PLP-dependent subgroup of the Radical SAM superfamily), and all HMM matches are to subgroups and/or families in that superfamily). The ‘Align to this family’ link opens an MSA of the chosen family with the query sequence. The results provided in Figure 2 show clearly that in the l-lysine 2,3-aminomutase family, the aspartate residues that bind the lysine substrate in all family members are replaced by a lysine and asparagine in GI:390523686. On the other hand, these lysine and asparagine residues are conserved throughout the glutamate 2,3-aminomutase family and are associated with binding the substrate, glutamate. Notably, a glutamate 2,3-aminomutase is ranked 18th in the BLAST hit list. Thus, this exercise allows the user to conclude that the annotation of GI:390523686 as a l-lysine 2,3-aminomutase is likely incorrect. Instead, the SFLD data provide evidence that this protein is more likely a glutamate 2,3-aminomutase. By extension, the close homologs to this protein sequence in the BLAST results from NCBI that are annotated as l-lysine 2,3-aminomutases are also likely to be incorrect.

### Studying sequence–function relationships using SSNs

Clicking the PLP-dependent subgroup (Figure 2) and downloading the PLP-dependent subgroup SSN, using an *E*-value cutoff of 1e^−^^85^, produces a ‘.XGMML’ file that can be imported into Cytoscape. Since each family is automatically colored differently by the functional assignment in the SFLD, the relationship between the query protein and the different families can be easily visualized. Compared to BLAST results, which simply list pairwise similarities between the query and other proteins, the network view allows simultaneous appreciation of additional dimensions of data, namely the similarities among all the other proteins as well as to the query. The network visualization also supports the suggested association between GI:390523686 and the glutamate 2,3-aminomutase family (Figure 2). Using the Cytoscape software, users can customize coloring of nodes, node shape, edge representation and many other properties using the network and attribute files downloaded from the SFLD. Cytoscape also supports generation of high-quality network figures, such as in Figures 1 and 2, for publication.

### Target selection

SSNs can also be used for target selection efforts, aimed at determination of new functions (e.g. substrate specificities) in a superfamily of interest [e.g. (35,44)]. In Figure 2, the presence of SSN clusters that are not assigned to a family (examples highlighted with black arrows) suggests there may be additional, perhaps unknown, reaction families in this subgroup. The network context facilitates studying these clusters. For instance, choosing all bacterial proteins (by the ‘Type of life’ attribute), that have DNA available or potentially meaningful genomic context [links to MicrobesOnline (45) and the SEED (46) are available] can help in pinpointing specific targets that may be useful for subsequent wet-lab experimentation.

## DISCUSSION

The SFLD database aims at providing a reliable resource of enzyme classification that integrates both sequence and functional criteria into a hierarchy designed to assign functional properties only at the level (superfamily, subgroup, family) at which conservation patterns and their associated functional features can be inferred with confidence. Thus, if a new sequence appears to have only those residues conserved that allow its assignment to a superfamily and subgroup, but not to a reaction-specific family, it is only assigned at the superfamily and subgroup levels. This practice is useful for avoiding misannotation of specific function from overly-simple inference of orthology (20). Based on careful curation of superfamilies and the utilization of tools that allow database expansion and updates, we strive to cover an increasing portion of the enzyme world. Specifically, integration of the SFLD with the powerful capabilities of networks (28) will allow us to expand the extended SFLD more quickly than can be achieved by manual curation. As the list of superfamilies in the extended SFLD grows, we invite groups working on those enzymes to contact us as we are interested in forming collaborations with experimentalists to improve our annotations.

While the classification model described here works well for the majority of data entered into the database, the complexity of structure–function mapping in some superfamilies can pose special challenges. For instance, there are chemical reactions that are catalyzed by residues in distinct subunits of heterodimers, complicating association of functional residues to sequence data. Other cases involve multidomain proteins that are not well accommodated by our current definition of EFDs. Hence, we continue to extend the database schema to adequately curate complex biological examples.

SSNs serve as a powerful tool for both curation and analysis. Nevertheless, several caveats for the use of SSNs are important to avoid their over- or too simple interpretation. For example, SSNs from the SFLD are created using full-length sequences. In some cases, the EFD is only a small segment of a multidomain protein. Thus, an edge in an SSN may represent the similarity between any (or all) regions of the sequences represented by the connected nodes, even parts outside the EFD. It is important for the users to identify the parts of sequences that contribute to the similarity scores to be sure they capture relevant features like active site motifs. This can be achieved, in part, by using thresholds for viewing the networks that are statistically significant, thereby ensuring some confidence in the underlying alignments on which the network connections are based [see (26) for further discussion of network validation and issues for their use]. Additional caveats of SSNs are described in the online documentation.

The future directions of the SFLD involve four main areas. First, the automated superfamily expansion protocol will be optimized and deployed. Second, we intend to facilitate function prediction for metagenomics research by placing these sequences of unknown function into the context provided by superfamily SSNs to aid their annotation (28). Third, we intend to supply more information about the genomic context, i.e. operons and/or nearby genes, of query proteins. This will aid identification of new pathways or involvement of query enzymes in bacterial operons. Last, we intend to make the chemical reaction information more integrated and searchable.

## FUNDING

The National Institutes of Health [grant number R01GM60595 to P.C.B], the National Science Foundation [DBI-0234768, DBI-0640476 to P.C.B.]; National Institute of General Medical Sciences [P41GM103311 to T.E.F.]. Support for recent expansion and improvements was also provided by the National Institute of General Medical Sciences [U54GM093342, P01GM07790 to P.C.B]. Funding for open access charge: National Institutes of Health [GM60595 to P.C.B.].

*Conflict of interest statement*. None declared.
